# P-Glycoprotein 1 Affects Chemoactivities of Resveratrol against Human Colorectal Cancer Cells

**DOI:** 10.3390/nu11092098

**Published:** 2019-09-04

**Authors:** Virginie Aires, Didier J Colin, Agnès Doreau, Attilio Di Pietro, Jean-Marie Heydel, Yves Artur, Norbert Latruffe, Dominique Delmas

**Affiliations:** 1 Université de Bourgogne Franche-Comté, F-21000 Dijon, France; 2 Bioactive Molecules and Health research group—Cancer and Adaptative Immune Response Team—INSERM Research Center U1231, F-21000 Dijon, France; 3 BioPeroxyl, EA 7270, F-21000 Dijon, France; 4 MicroPET/SPECT/CT Imaging Laboratory, Centre for BioMedical Imaging (CIBM), University Hospitals and University of Geneva, 1211 Geneva 4, Switzerland; 5 Institut de Biologie et Chimie des Protéines, UMR5086 CNRS-Université Lyon 1, F-69000 Lyon, France; 6 Centre des Sciences du Goût et de l’Alimentation, UMR 6265 CNRS/1324 INRA, 9 Boulevard Jeanne d’Arc, F-21000 Dijon, France

**Keywords:** resveratrol, colon cancer, ABC transporters, sensitivity, transport

## Abstract

Resveratrol has been proposed to prevent tumor growth and the different steps of carcinogenesis; nevertheless, these biological effects are sometimes discordant between different cell types. Several hypotheses and works have suggested that the metabolism of resveratrol could be at the origin of a different cellular response. We show here, using colorectal tumor cell lines, that the biological effects of RSV result mainly from its carriage by carriers of the superfamily of ABC transporter, i.e., P-gP, MRP, or BCRP. Using cell lines overexpressing these different transporters, we have been able to highlight the importance of P-gP in the response of cells to RSV. These results were confirmed by invalidating the gene coding for P-gP, which restored the sensitivity of colorectal cells resistant to the polyphenol. Subsequently, the status of P-glycoprotein expression is an important element to be taken into consideration in the cytotoxic activity of resveratrol in colorectal cancer cells.

## 1. Introduction

For many decades, various investigations have searched to identify new compounds which are able to induce apoptosis without deleterious effects on the normal cells. Among these compounds, dietary polyphenols appeared to have great interest in anticancer strategies, notably by their pro-apoptotic potential. Numerous studies have reported interesting properties of a polyphenol, *trans*-resveratrol (RSV), as a chemopreventive agent against various pathologies—i.e., vascular diseases, cancer, viral infection, or neurodegenerative processes (see for review [1,2,3]. In fact, resveratrol (RSV or *trans* 3,5,4′ trihydroxystilbene) is a polyphenolic antifungal phytoalexin found in various food products, with particularly high levels in grape skin (50–100 µg/g). In various in vitro and in vivo models, this polyphenolic compound has proved to be able to retard or prevent the stages of carcinogenesis [4]. This protective effect could be related to the RSV ability to arrest the cell cycle or to trigger tumor cell death mainly by apoptosis [5,6]. Furthermore, in chemotherapy, it appears that RSV can sensitize colon cancer cells to 5-fluorouracil, which is a classic drug used in colorectal and hepatoma chemotherapy. Indeed, it was reported that RSV can exert a synergistic effect with this drug to inhibit hepatocarcinoma and colon carcinoma cell proliferation by the induction of apoptosis [5,7,8,9]. Moreover, we have shown that a part of RSV’s action involves its transport by an active process implying raft raft-mediated endocytosis in colon cancer cell lines. Indeed, the polyphenol accumulates into lipid rafts and recruits various signaling proteins especially integrins and MAP kinases to induce early signaling cascades leading to apoptosis [10]. Interestingly, this mechanism is only produced in cancerous cells and not in normal cells which are protected from RSV action. Nevertheless, various cancer cells react more or less differently to the action of RSV, in particular colorectal cells [9]. The resistance of cancer cells to cytotoxic molecules is often due to the overexpression of membrane transporters belonging to ATP-binding cassette (ABC) superfamily. Among these transmembrane proteins, the cellular protein P-glycoprotein (P-gP/MDR1/ABCB1), the multidrug resistance proteins (MRPs), and the breast cancer resistance protein (BCRP/ABCG2) mediate classic multidrug resistance (MDR) in cancer cells by functioning as an energy-driven efflux pump. In this way, these efflux proteins confer resistance to a variety of natural product type drugs. Some studies have shown that ABC transporters could transport the resveratrol such as BCRP at pH 6.0 but not at pH 7.4 [11], and MPR2 [12]. Cooray et al. have also shown in BCRP-expressing cells, that resveratrol treatment was able to accumulate BCRP substrates [13]. Nevertheless, the potential link between the ability of RSV to induce a differential action in colorectal cancer cell line and the overexpression of main transporters remains to be explored. Moreover, the clinical studies of resveratrol are much contrasted due to various parameters including the number of participants, health status of the gut microbiota, age, gender, lifestyle, dose, administration medium, and type of administration, and the modulation of pharmacokinetics of RSV which present a poor bioavailability [14]. For this last reason, it could be interesting to increase the amount of RSV in tissue by suppressing MDR activities. In the present study, we show for the first time, the link between the differential expression of the P-glycoprotein (P-gP/MDR1/ABCB1), of the multidrug resistance proteins (MRP1 and 2) and of the breast cancer resistance protein (BCRP) in four colorectal cell lines (SW480, SW620, HT29, and HCT116) and the ability of these cells to efflux RSV. By using specific inhibitors of ABC transporters and a decrease of temperature, we demonstrate their involvement in RSV transport and their impact on RSV biological action. Moreover, the used of specific cell lines overexpressing each of the transporters studied, we have identified the importance of MDR1 in the RSV transport and in its cytotoxic action. These results are comforted by MDR1 silencing which restores a cytotoxic effect of RSV against colorectal cells.

## 2. Material and Methods

### 2.1. Cell Lines

SW480, SW620, HT29, and HCT116 human colon carcinoma cell lines were obtained from the American Tissue Culture Collection (ATCC, Rockville, MD, USA). SW480, HCT116, HT29, and SW620 cells were cultured in Eagle’s minimum essential medium, complemented with 10% (v/v) fetal calf serum (Sigma-Aldrich, Saint Quentin Fallavier, France). Mouse embryonic fibroblast NiH3T3, human embryonic kidney cells HEK 293, and baby hamster kidney cells BHK 21 were maintained in Eagle’s minimum essential medium complemented with 10% fetal calf serum, as their stably-transfected clones NiH3T3-MDR1, HEK293-MRP2, BHK21-MRP1, and HEK293-BCRP. Transfected clones were generated and kindly provided by Dr. Attilio Di Pietro. Resveratrol treatments were performed by incubating cells for indicated times at specified concentrations in medium containing 0.1% ethanol.

### 2.2. Drugs, Chemical Reagents, and Antibodies

[^3^H]-*trans*-resveratrol (specific activity: 74 GBq/mmol) labeled in *ortho* and *para* of benzenic rings was prepared by Amersham (Aulnay sous Bois, France). All chemicals were obtained from Sigma-Aldrich (Saint Quentin Fallavier, France) unless specified. MDR1 antibody was purchased from Thermofisher Scientific, BCRP and MRP2 antibodies were from Cell Signaling Technologies and MRP1 was obtained from Abcam. Horseradish peroxidase-conjugated secondary antibodies and anti-β-actin were obtained from Sigma-Aldrich.

### 2.3. Resveratrol Efflux Measurement

Resveratrol efflux was examined by incubating confluent cells in 6-well plates over 10 min with 30 µM tritiated resveratrol in complete medium at 37 °C. Cells were also preincubated for 10 min with ABC transporter inhibitors such as verapamil (20 µM), cyclosporin (20 µM), and indomethacin (20 µM). At the end of the incubation period, the labeled medium was removed and the cells were washed two times with PBS. Fresh medium (37 °C or 4 °C) was then quickly added to allow the cells to efflux tritiated resveratrol from 1 to 360 min. At the end of each time point, the cells were washed and lysed in NaOH 0.1 M, Na_2_CO_3_ 2%, SDS 0.1%. The cell homogenates were transferred into flasks and cell-associated radioactivity was counted in a liquid scintillation analyzer. We evaluated 60 min area under curves (AUC) by integrating exponential regression curves of [^3^H]-resveratrol time course accumulation between 0 and 60 min.

### 2.4. Cytotoxicity Assays

Cell lines were seeded into 96-well plates. After 24 h of culture, the cells were treated with increasing concentrations of resveratrol for 72 h. All control and treated cells received the same volume of ethanol (0.1%). After time of treatment, cells were harvested and the toxicities were evaluated by crystal violet staining. Briefly, after treatments cells were washed with PBS 1X, stained for 5 min with a crystal violet solution (0.5% (w/v) crystal violet in 25% (v/v) methanol), and then gently rinsed with water. Absorbances were read at 540 nm with a Biochrom Asys UVM 340 spectrophotometer, after the extraction of the dye by 0.1 M sodium citrate in 50% ethanol. The inhibitory concentrations 50% (IC_50_) were assessed by crystal violet staining after 72 h of treatment. IC_50_ values were calculated by a four-parameter non-linear regression with GraphPad Prismversion 6 software (San Diego, California, CA, USA). In some experiments, after 24 h of seeding, cells were treated with resveratrol (30 µM) for 48 h before crystal violet staining. All results were expressed as percentages of control values (vehicle alone).

### 2.5. Apoptosis Identification

Cells were seeded into 6-well plates 24 h before treatments. Then cells were untreated (Co, vehicle) or treated with either RSV alone (10, 30, or 50 µM). After 72 h of treatments, supernatants of each well were collected and the adherent cells were recovered by trypsinization with 1 mL of trypsin/EDTA solution. Suspensions containing supernatant and trypsinized cells were washed with PBS 1X, stained with 1 µg/mL Hoechst 33342 (Sigma-Aldrich) for 15 min at 37 °C and then mounted onto glass slides. The fluorescence was detected using an epi-fluorescence microscope (Axioskop Leica) using the DAPI filter (λexc: 365 nm and λémi: 397 nm). Percentages of viable (intact nuclei) and apoptotic cells presenting typical nuclear chromatin condensation and fragmentation were determined by analyzing 300 cells from randomly selected fields.

### 2.6. Western Blotting

Cells were lysed in RIPA buffer (50 mM Tris, 150 mM NaCl, 0.5% NaDeoxycholate, 1% NP40, 2 mM EDTA, 50 mM NaF, 100 µM PMSF; pH 8) containing complete ultra-protease/phosphatase inhibitor (Roche). Proteins (40 µg) were resolved by SDS-PAGE and transferred to nitrocellulose membranes (Amersham). Blots were then saturated in 5% milk (1 h at room temperature) before overnight incubation at 4 °C with specific primary antibodies. All primary antibodies were diluted at 1:1000 in 5% w/v non-fat milk or 5% BSA. Primary antibodies were detected using horseradish peroxidase (HRP)-conjugated appropriate secondary antibodies (Cell Signaling Technologies, Danvers, MA, USA) followed by exposure to ECL (Santa Cruz Biotechnology, Dallas, TX, USA). A signal was acquired with a ChemiDoc™ XRS+ imaging system (Biorad) and blots were analyzed with Image Lab™ software 5.1.2 (Biorad, Marnes-la-Coquette, France).

### 2.7. RNA Extraction and Quantitative PCR Analysis

All treatment groups from a same experiment were harvested at the same time and total RNA was prepared using Trizol Reagent (Invitrogen) according to the manufacturer’s instructions. All of the samples for comparison were reverse transcribed from the same reverse transcription (RT) mixture. To generate cDNA, total cellular RNA was extracted with TRIzol^®^ RNA Isolation Reagent (Ambion). RNA (500 ng) was reverse-transcribed into cDNA using M-MLV reverse transcriptase, random primers and RNAseOUT inhibitor (Invitrogen). cDNA was quantified by real-time PCR with the Power SYBR Green PCR Master mix (Applied Biosystems; Warrington, UK) on a 7500 Fast Real-Time PCR detection system (Applied Biosystems, Foster City, CA, USA). Relative mRNA levels were determined by the ΔΔCt method and normalized to the expression levels of housekeeping genes used as internal controls, i.e., human β-actin (*Actb*) and glyceraldehyde-3-phosphate dehydrogenase (GAPDH). Results are expressed as means ± standard error (SE) from duplicate PCR determinations of triplicate 6-well plates from three independent experiments for each set of conditions tested. The following primer sequences were used: *hMDR1:* sense 5′-CTTGGCAGCAATTAGAAC-3′, antisense 5′-TCAGCAGGAAAGCAGCAC -3′; *hMRP1:* sense 5′-GGATCATGCTCACTTTCTGG-3′, antisense 5′-AAGTGATGTCACGAAACAGGTC-3′; *hMRP2:* sense 5′-ACAGAGGCTGGTGGCAACC-3′, antisense 5′-ACCATTACCTTGTCACTGTC-3′; *hBCRP:* sense 5′-TGGCTTAGACTCAAGCACAGC-3′, antisense 5′-TCGTCCCTGCTTAGACATC-3′; *hActb:* sense 5′-AGAGCTACGAGCTGCCTGAC-3′, antisense 5′-AGCACTGTGTTGGCGTACAG-3′; *h GAPDH:* sense 5′-GCCATCAATGACCCCTTCATT-3′, antisense 5′-TTGACGGTGCCATGGAATTT-3′.

### 2.8. Transient Transfection

For small interfering RNA (siRNA)-mediated knockdown of MDR1, SW480 cells were reverse-transfected with 10 nM of either the targeting siRNA Silencer^®^ Select (validated siRNA, #AM51331, assay ID 4123, Invitrogen) or negative control siRNA (Silencer™ Negative Control No. 1 siRNA, Invitrogen #AM4611) using Lipofectamine RNAiMax as the transfection reagent (Invitrogen) for 24 h. Fresh or resveratrol-containing media were then added to cells for 72 h before crystal violet staining and assessment of cytotoxicity, or analysis of knockdown efficiency by western blotting.

### 2.9. Statistical Analysis

Statistical analysis of data was carried out with Prism GraphPad6.0 Prism Software. The significance of the differences between mean values was determined by a one-way ANOVA with Holm–Sidak correction or with the Student’s test. *p*-values < 0.05 were considered significant (* *p* < 0.05, ** *p* < 0.01, and *** *p* < 0.001).

## 3. Results

### 3.1. Differential Antiproliferative Responses of Colon Cancer Cells towards Resveratrol

**Figure 1:**
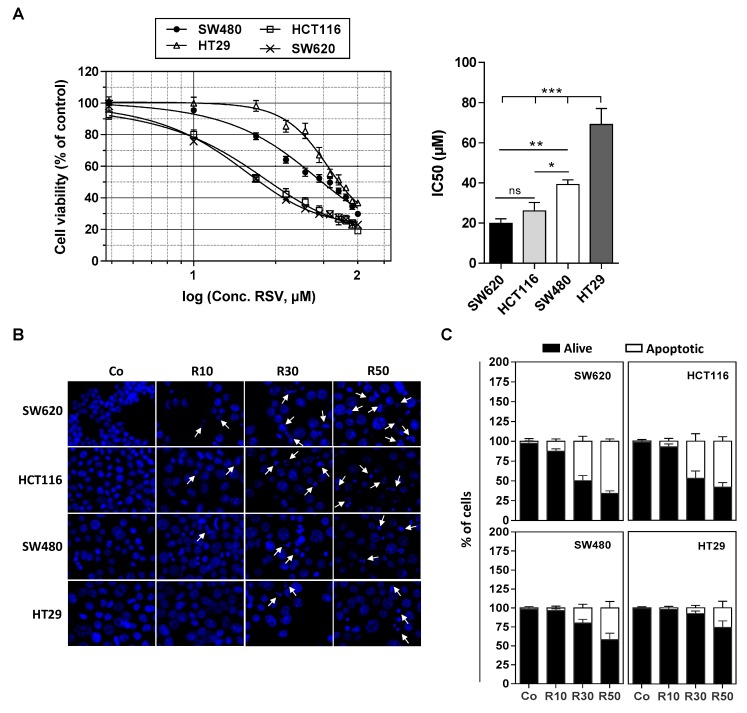
Differential sensitivity of human colorectal cancer cell lines to resveratrol. (**A**) After 24 h of culture, colon cancer cell lines SW480 and their metastatic phenotype SW620, HCT116, and HT29 were left untreated (Co, 0.1% ethanol) or were treated with increasing concentrations of resveratrol (RSV; concentrations ranging from 1 to 100 µM) for 72 h. Cell survival was expressed as a percentage of control cells (vehicle alone) after crystal violet staining. RSV concentrations were log_10_ transformed and inhibitory concentrations 50% (IC_50_) were determined after non-linear regression curve fitting and then plotted using GrapPad Prism software (left panel). Data are mean ± S.D. of three independent experiments each containing six replicates per concentration (right panel). *P*-values were determined by one-way ANOVA followed by a Holm–Sidak post-hoc test. * *p* < 0.05, ** *p* < 0.01, and *** *p* < 0.001. (**B**) Cells were left untreated (Co) or treated with RSV at 10, 30, or 50 µM for 72 h before nuclear staining with Hoechst 33342. Representative fluorescence microscopy images of three independent experiments are shown, with white arrows indicating typical condensed/fragmented apoptotic nuclei. Original magnification: ×40. (**C**) Quantification of both alive and apoptotic cells was performed by counting intact and fragmented nuclei in 300 cells per cell line (randomly selected fields) with Image J software. Data were expressed as mean percentage ± S.D.

We have previously shown that various tumoral cells responds differently to apoptosis induced by RSV and its metabolites (i.e., RSV-sulfate, RSV-glucuronide) especially against colon carcinoma cells [9,15]. We confirmed by a cytotoxic assay with crystal violet on numerous colon cancer cell lines exposed to RSV treatment during 72 h with various concentrations (1 to 100 µM) that they present different sensitivities in a dose-dependent manner (Figure 1A). Indeed, as revealed by cytotoxic curves, we observed that HT29 cell line presents the highest resistance against RSV, whereas conversely the lines HCT116 and SW620 appear more sensitive to the action of RSV (Figure 1A). These results are supported by the determination of the IC_50_ (concentration inhibiting 50% of the tumor proliferation) where the HT29 and SW480 cells have an IC_50_ around of 40 and 70 µM respectively, while the SW620 and HCT116 cells an IC_50_ around 20 and 25 µM respectively (Figure 1B). These results are consistent with the ability of RSV to differentially induce apoptosis in these cells (Figure 1B,C). Indeed, as we showed previously, when cultured in the presence of RSV, colon carcinoma cells underwent apoptosis (Figure 1A). Cell staining with Hoechst 33342 demonstrated that RSV induces an increase in the nucleus size that preceded the appearance of characteristic apoptotic changes, i.e., the condensation and fragmentation of the nuclear chromatin (Figure 1B). This process can be quantified and we confirmed as in Figure 1A the differential response of colon carcinoma cell lines to RSV since we observed that a game range of RSV (10–50 µM) induces a most important apoptosis in SW620 and HCT116 cell lines in a concentration dependent manner, which is strongly reduced in SW480 and HT29 cells (Figure 1C). 

### 3.2. Differential Expression of Main ABC Transporters in Human Colon Carcinoma Cells

**Figure 2:**
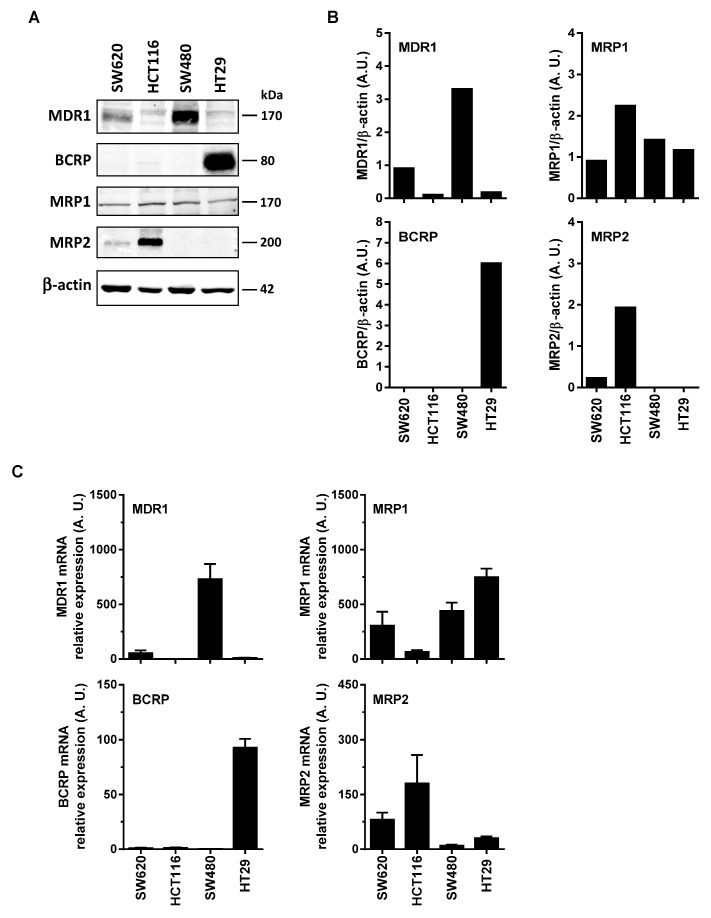
Differential expression of ABC transporters in human colorectal cancer cell lines. (**A**) Cells were seeded in T25 cm^2^ flasks and cultured in complete media until confluency was reached and before protein extraction and western blot analysis with specific anti-ABC transporters primary antibodies. Representative immunoblots from three independent experiments are shown. β-actin was used as a loading control. (**B**) Densitometry quantification of representative blots. (**C**) In the same experimental conditions as in (A), total RNA were extracted from confluent cells and ABC transporters genes were analyzed by RT-qPCR. Histograms represent mean relative expression (A.U., arbitrary units) ± S.D. of three independent experiments each performed in triplicate per condition. The efflux of resveratrol is mediated by active mechanisms in human colon carcinoma cells.

Some reports have shown that RSV transport and its metabolites could involve the ABC transporters. Indeed, ABC transporters could transport the resveratrol such as BCRP at pH 6.0 but not at pH 7.4 [11], and MPR2 [12]. Cooray et al. have recently shown in BCRP expressing cells, that resveratrol treatment was able to accumulate BCRP substrates [13]. These transports of RSV and its metabolites by an ABC-dependent mechanism could be at the origin of the differences of sensitivity of the colonic tumor cell lines to the RSV action. In order to explore the possible correlation between colon cancer cells responses toward RSV and the enhancement of its efflux, we first measured the basal level of the main ABC protein expressed in the colon carcinoma cell lines studied. Immunoblotting of colon cancer cells revealed that more resistant cells, SW480 and HT29 overexpressed significantly MDR1 and BCRP respectively (Figure 2A,B). Except for MRP1 expression which is stable for all the colorectal cell lines tested (Figure 2A), the most sensitive metastatic colon cancer cell line, SW620, did not express BCRP, very slightly expressed MRP2, and a little more MDR1. These differences in the protein expressions are also found in the gene expressions between the tumor cell lines (Figure 2C). Indeed, RT-QPCR analysis indicates that, as for immunoblotting, SW480 cell line has the highest amount of MDR1 mRNA, whereas for HT29 it is as above BCRP which is strongly overexpressed compared to other cell lines (Figure 2C). Similarly for MRP2, the amount of mRNA is the largest in the HCT116 cell line.

In order to explore the possible correlation between colon cancer cells responses toward resveratrol and the enhancement of its efflux, we measured the intracellular tritiated RSV content in the different colon cancer cell lines already described for their sensitivity to resveratrol-induced tumoral cell proliferation inhibition and apoptosis (Figure 1A,B).

**Figure 3:**
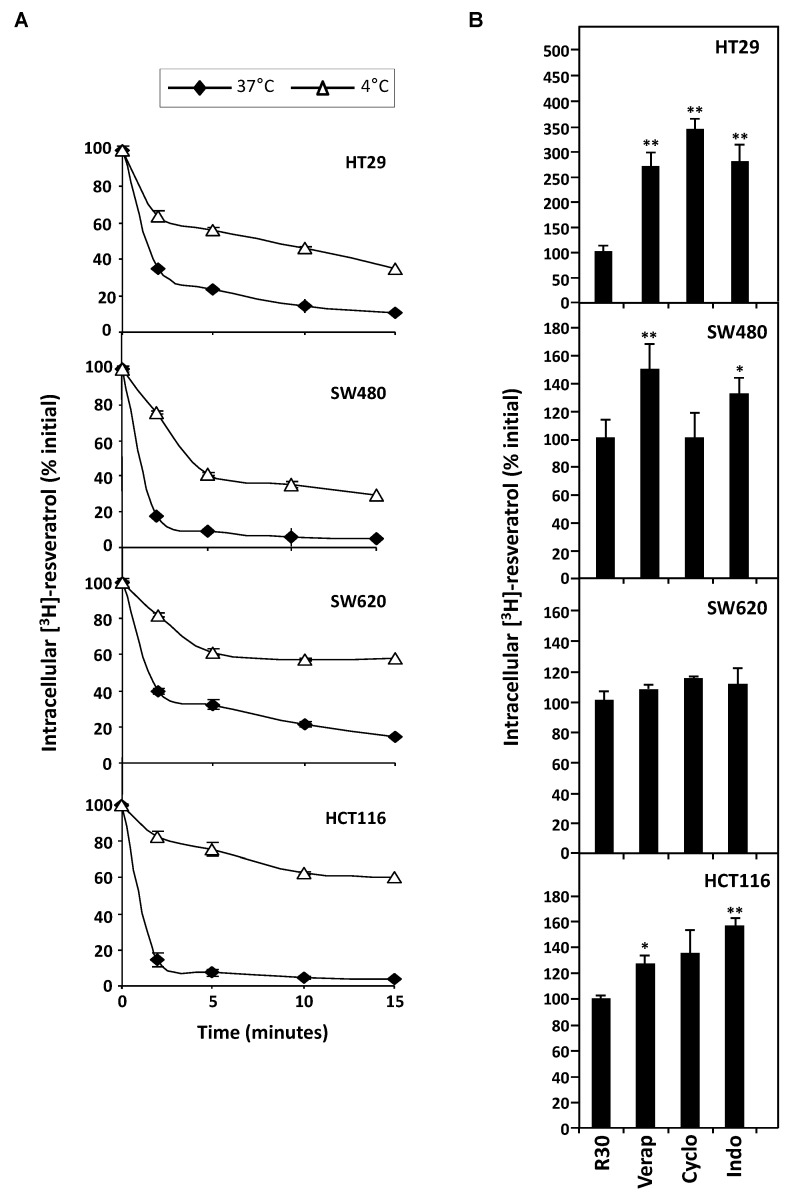
Involvement of active process in the efflux of tritiated resveratrol in human colon carcinoma cells. (**A**) SW480, HT29, SW620, and HCT116 cells were submitted to time course measurement of [^3^H] resveratrol efflux. Cells were grown in 6-well plates until confluence and treated for 10 min with 30 µM of [^3^H] resveratrol (uptake phase). Cells were then quickly washed with PBS 1X and [^3^H] resveratrol was allowed to efflux in fresh medium at 37 °C and 4 °C for indicated times. Cells were lyzed and radioactivity levels were determined and plotted as percentages of initial levels measured after uptake phase. Results are statistically different between 37°C and 4°C efflux experiments with *p*-value at least < 0.05 by Student’s test. (**B**) Confluent colon cancer cells were pretreated for 10 min with efflux pumps inhibitors, 20 µM verapamil (Vera), 20 µM cyclosporine A (Cyclo), or 20 µM indomethacin (Indo). They were then submitted to 10 min of [^3^H] resveratrol uptake, washed and efflux phase was conducted for 5 min in 37 °C fresh medium. Resveratrol levels are expressed as percentages of intracellular radioactivity in untreated cells. Data are means ± SD (n = 3) of a representative experiment among three independent ones. * *p* < 0.05 and ** *p* < 0.01 by Student’s test.

RSV efflux experiments were realized after the 10 min. of uptake period, which is the time to reach a maximal accumulation level [10] and with 30 µM concentration which induces a marked antiproliferative and proapoptotic effect without cell damage [6,10,16]. After the medium had been replaced by non-radioactive medium, the amount of [H^3^]-resveratrol present in the tumoral cell lysates was determined as a function of time. These experiments reveal that all colorectal cancer cell lines are able to efflux RSV to varying degrees during the first minutes at 37°C (Figure 3A). Interestingly, the most sensitive SW620 cell line retains RSV during longer times. In order to specify the mechanism of RSV efflux, we have tested the effect of temperature on the transport of polyphenol. We observed that [H^3^]-resveratrol accumulation in colon carcinoma cells was significantly higher at 4°C than at 37 °C, which was more significant with all cell lines tested and more particularly with HCT116 cells (Figure 3A). Analyzing the % efflux by studying the efflux curves of the Figure 3, the rate of efflux in % of resveratrol efflux per min shows that the decrease of temperature to 4 °C reduces, in all tested cell lines, the intracellular [H^3^]-resveratrol rate (Table 1). These delays in RSV accumulation suggest a probable active transport of the polyphenol. Therefore, to confirm the implication of transporters, we conducted resveratrol efflux analysis in the presence of different MDR and/or MRP inhibitors. Our results show that the tested inhibitory drugs, mainly those who have the largest action spectrum—like verapamil, cyclosporine, and indomethacin—are able to inhibit significantly RSV efflux (Figure 3B). It highlights that overexpression of BCRP and MDR transporters underlies HT29 and SW480 cells resistance to RSV. Indeed, the release of RSV is 2.5- to 3.5-fold inhibited by these drugs in HT29 cell line and 1.5-fold with verapamil and 1.8-fold with indomethacin in SW480 (Figure 3B). Very interestingly, the use of transporter inhibitors did not significantly change RSV uptake in the SW620 tumoral cell lines. Other ABC protein inhibitors enhance resveratrol retention in all cell lines (orthovanadate, ouabain, quinine, probenicid, quercetin). These results reinforce our hypothesis of an active pump involvement in the RSV efflux to impact its biological action.

### 3.3. Stable Overexpression of MDR Responsible ABC Transporters Mediate Resveratrol Efflux

**Figure 4:**
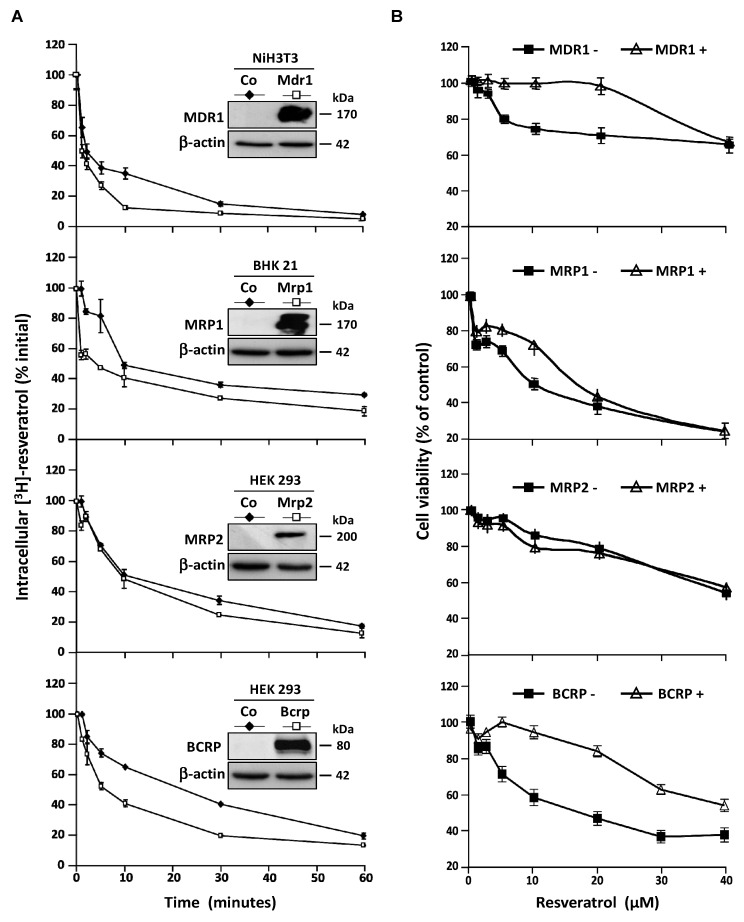
Resistance of colon cancer cell lines to resveratrol involves ABC transporters. (**A**) Control NiH3T3, BHK 21, HEK 293, and respective overexpressing clones NiH3T3/MDR1, BHK 21/MRP1, HEK 293/MRP2, and HEK 293/BCRP were submitted to time course measurement of [^3^H] resveratrol efflux. Cells were grown in 6-well plates until confluence and treated for 10 min with 30 μM of [^3^H] resveratrol (uptake phase). Cells were then quickly washed with PBS and [^3^H] resveratrol was allowed to efflux in fresh medium at 37°C for indicated times. Cells were lyzed and radioactivity levels were determined and plotted as percentages of initial levels measured after uptake phase. Inserts are western blot evaluation of ABC transporters proteins levels in control cells against stably transfected ones. Data are means ± SD (*n* = 3) of a representative experiment among three independent ones. Results are statistically different between control and ABC transporters overexpressing cells with *p*-value at least < 0.05 by Student’s test. (**B**) Dose–response curves to resveratrol in control and stably transfected cells after 72 h of treatment. Cell viability was expressed as percentage of control (vehicle alone). Data are mean percentage ± SD of three independent experiments each containing six replicates per condition.

These observations led us to characterize the transporters involves in resveratrol efflux by using four stably transfected cell lines who overexpressed the MDR1, MRP1, MRP2, and BCRP plasma membrane ABC transporters respectively. As previously, to compare the ABC transporters overexpression effects, we measured the intracellular [H^3^]-resveratrol rate during the time. In this way, we calculated the area under curve (AUC) between 0 and 60 min on exponential regression models of intracellular RSV level in the different cell lines (Figure 4A). We demonstrated that both MDR1 and BCRP are the most potent RSV carriers, their overexpression leading to 1.7-fold inhibition of RSV accumulation (Figure 4A). An early slight effect of MRP1 was observed while MRP2 was the less potent RSV carrier compared to the other one since the AUC was only diminished 1.2-fold between control cells and MRP2 overexpressing one (Figure 4A). In accordance with the RSV efflux studies, the cell lines overexpressing MDR1 and BCRP proteins show a resistance to antiproliferative RSV effect to compare control cell lines (Figure 4B). The cell viability level was maintained for a RSV game range of 1–40 µM, after this critical concentration, there are no differences between the cell lines overexpressed or not the ABC transporters.

### 3.4. MDR1 Silencing Expression Cells Enhances the Resveratrol Antiproliferative Activity

**Figure 5:**
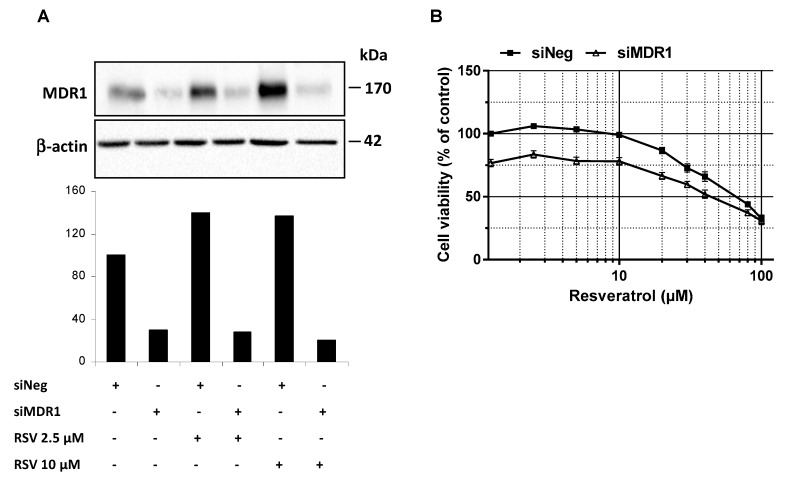
Inhibition of ABC transporters activity or expression restores colon cancer cell lines sensitivity to resveratrol. (**A**) SW480 cells were transiently transfected with negative control siRNA (siNeg) or MDR1 siRNA (siMDR1) for 24 h before treatment of cells for 72 h with or without the indicated concentrations of resveratrol. Thereafter, proteins were extracted and MDR1 protein expression levels in experimental conditions were analyzed by western blot. A representative blot and corresponding densitometry quantification from three independent experiments are shown. (B) SW480 cells were seeded in 96-well plates and transiently transfected with negative control siRNA (siNeg) or MDR1 siRNA (siMDR1) for 24 h before treatment of cells for 72 h with increasing concentrations of resveratrol. After treatments, cells were washed with PBS and cell viability was then assessed after crystal violet staining. Results were expressed as percentages of control (vehicle alone) and are mean ± SD of three independent experiments each containing six replicates per condition.

In addition to RSV transport in colon cancer cells, we investigated whether MDR1 silencing in SW480 colon cancer cells improves RSV antiproliferative properties. Firstly, we have found that the use of a wide ABC inhibitor—i.e., cyclosporine A (20 µM)—increases antiproliferative activity of RSV as compared to the control in SW480 colorectal cancer cells. Secondly, in order to highlight that resistance to resveratrol was reversible by inhibiting MDR1 in tumoral colorectal cells, we have knockdown MDR1. Indeed, MDR1 silencing by a transient transfection with a siMDR1 showed a strong decrease of the basal level of MDR1 in SW480 and counteracted the MDR1 overexpression induced by RSV (2.5 and 10 µM) as shown by the MDR1 protein expression (Figure 5A). Moreover, MDR1 silencing in resistant SW480 colorectal cells went along with a significant increase of cell survival under RSV treatment (Figure 5B). Altogether, data demonstrate that MDR1 is essential for RSV antiproliferative activities.

## 4. Discussion

There is compelling evidence that RSV can act as a chemopreventive agent or a chemotherapeutic drug against various cancers through the regulation of multiple therapeutic targets [3] without effect on normal cells [10,17]. Nevertheless, there is great variability in the biological response of cancer cells to polyphenol. Various studies demonstrated the importance of RSV metabolization to produce active metabolites [15,18], but many other endogenous or exogenous factors may be involved in the differential responses that can be observed. In the present report, we studied the impact of various colorectal cancer cell lines expressing each different membrane transporter involved in drug efflux. We found that RSV is differently treated depending on the cell lines used, the ones that effluent the faster, and most importantly the RSV are those that are the least sensitive to the cytotoxic action of polyphenol. The study of the expression of ABC transporters allowed to correlate the rate of efflux of RSV and the sensitivity of the lines to the expression of MDR1 and BCRP. Among these main transporters, the MDR1 silencing expression has shown that RSV action on colorectal cancer cell lines is dependent of its expression.

Resistance of cancerous cells to cytotoxic drugs is often thought to be a major cause of the failure of chemotherapeutic treatment of malignant neoplasms. Multidrug resistance (MDR) can be present before treatment (intrinsic) or can develop during chemotherapy (acquired) and it may extend to structurally and functionally unrelated drugs. Colon cancer is regarded to be intrinsically resistant to chemotherapy. This chemoresistance can be due to various factors such as a lack of induction of apoptosis resulting from a decrease in the activation of cell death cascades or an overexpression of antiapoptotic proteins such as Bcl-2, or an increase of xenobiotic metabolism leading to its elimination or an increase in the efflux of the anticancer drug. In our study, we have used four colorectal cancer cell lines: SW480 cell line, which was established from a primary adenocarcinoma of the colon and which is positive for expression of c-myc, K-ras, H-ras, N-ras, myb, sis, and fos oncogenes and for the p53 protein; SW620 which was derived from a metastasis of the same tumor from which the SW480. It was isolated from the tissue of a 51-year-old Caucasian male (blood group A, Rh+) and as SW480 this line is positive for expression of c-myc, K-ras, H-ras, N-ras, Myb, sis, fos, and p53 oncogenes. The HT-29 line was isolated from a primary tumor in 1964 using the explant culture method and the line is positive for expression of c-myc, K-ras, H-ras, N-ras, Myb, sis, and fos oncogenes. The p53 antigen is overproduced. The last colorectal cell line is HCT116 which was isolated from a colorectal carcinoma and which has a mutation in codon 13 of the ras proto-oncogene. In view of their oncogenic status and more particularly the level of expression of the p53 protein, it does not seem that this status plays in our model which is in agreement with other articles showing, in particular, that the level of expression of the protein p53 does not influence the response of colorectal tumor cells to resveratrol [19] and in other cancer models [20,21]. Some reports have shown in the colon cancer models that MDR is often mediated by expression of drug transporters that catalyze an energy-dependent efflux of drugs, thus reducing intracellular drug concentration. Classical MDR was initially related to overexpression of the ATP-binding cassette (ABC) superfamily of transmembrane transporters such as the cellular protein P-glycoprotein (P-gP/MDR1), the multidrug resistance proteins (MRPs) and the breast cancer resistance protein (BCRP). P-gP mediates classic MDR in cancer cells by functioning as an energy-driven efflux pump. Increased levels of P-gP have occasionally been observed in malignancies following exposure to chemotherapeutic drugs and high levels of P-gP expression have been found to predict disease relapse in Duke’s stage B2 (pT_3_N_0_M_0_) colon cancers [22]. Like P-gP, MRPs can confer MDR by decreasing the intracellular drug concentration, such as MRP1 (ABCC1) which confers resistance to a variety of natural product type drugs. BCRP, a member of the ABCG half-transporter subfamily [23], has a drug resistance profile similar though not identical to P-gP. In this way, various studies have reported a potential action of RSV to decrease P-gP expression [24] and subsequently to enhance the activity of various anticancer drugs [25,26,27]. Given the rapid efflux of resveratrol colonic tumor cells, the question may arise whether the main metabolites of resveratrol are supported or not by the latter or if only the aglycone molecule is concerned. Furthermore, it is also important to consider whether RSV is administered alone or in combination with other phenolic substances or other agents capable of blocking the MDR phenomenon. Indeed, various studies have shown that natural polyphenols or their synthetic analogs can inhibit the MDR transporters responsible for chemotherapy resistance, including P-gP, MRP1, and BCRP [28]. Indeed, some polyphenols, such as the epigallocatechin gallate (EGCG), are well-known as P-gP blockers that downregulate P-gP and BCRP but do not inhibit MRP1 in a tamoxifen resistant breast cancer cell line [29]. Other polyphenols with hydrophobic groups like prenyl substituents may be promising future candidates for MDR reversal agents. For example, quercetin was able to inhibit the ATPase activity of MRP1 and 2 [30]. This study reveals in particular that the total number of methoxylated moieties, the total number of hydroxyl groups, and the dihedral angle between the B- and C-ring are crucial for MRP1 inhibition, unlike MRP2, where only the presence of a flavonol B-ring pyrogallol group seems to be an important structural characteristic.. Moreover, it is also very important to take into account the balance between influx and efflux. Indeed, we have demonstrated in the colorectal cancer lines studied in this report that RSV penetrated through an endocytosis-raft-dependent mechanism that conditioned the early activation of many signaling pathways (e.g., ERK, JNK kinases activation) to be successful to the phenomenon of apoptosis. However, some other polyphenols can also present an influx through the activity of P-gP [10,31]. Recently, a study of structure–affinity relationship of flavonoids as substrates of P-gP has highlighted that the presence of 4′-OCH_3_, 3′-OCH_3_ and the absence of 3′-OH, 3-OH, and 4′-OH in the core structure of flavonoids proved to be favorable for the affinity of flavonoids to P-gP [32]. Thus, the use of complex mixtures of polyphenols or their metabolites and the competition phenomena between influx and efflux must be taken into account in studies showing differences in biological activities of bioactive natural molecules. Nevertheless, these parameters are not the only things to consider. Indeed, we were able to show in this study that the combination of RSV with pharmacological inhibitors conventionally used clinically such as verapamil was able to block the efflux of RSV in a very efficient way and thus allowed to increase the effectiveness cytotoxic effect of RSV. Also, although RSV has been able to show real preclinical efficacy in many animal models, clinical efficacy remains to be demonstrated. In view of these early results in colorectal cancer cells, the combined use of RSV with one of these pharmacological inhibitors of MDR or MRP would counteract the low bioavailability of polyphenol and increase its effectiveness. Moreover, Mieszala et al. have recently shown that RSV reduced the expression of ABC subfamily B member 1, annexin A1 (ANXA1) and thioredoxin (TXN) genes and the proteins encoded by these genes which are associated with MDR [33]. These results suggest that RSV could reduce the resistance of cancer cells by affecting the expression of a number of the genes and proteins associated with MDR. Besides, our results indicate that the basal levels and activities of ABC transporters and the type of ABC transporters can be important to predict biological action of RSV.

## 5. Conclusions

Drug resistance accounts for poor treatment outcomes and tumor relapse. Resistance to conventional therapeutics is partly attributed to the enhanced activity of cellular efflux transporters leading to reduced intracellular levels of drugs and as a consequence, to tumor cells insensitivity to a wide range of therapeutical agents. This multiple drug resistance (MDR) phenotype is supported by the overexpression of members of the ATP binding cassette (ABC) transporter superfamily, which can pump out of cells multiple substrates. In this study we showed that ABC transporters i.e. P-gP and BCRP are the main efflux pumps responsible for colorectal tumor cells insensitivity toward resveratrol, a naturally occuring polyphenol with potential anticancer activity. Furthermore, we showed that resveratrol resistance could be reversed by the pharmacological inhibition or silencing of transporters. Hence, modulating the expression levels of these transporters might be of valuable interest to increase resveratrol bioactivity in a tumoral context.

## Figures and Tables

**Table 1:** Resveratrol efflux rates in colon cancer cell lines as a function of temperature

Tumor Cell Line	Vi = % of Resveratrol Efflux . min^−1^	
	37 °C	4°C
HT29	5.9	4.3
SW480	6.3	4.7
SW620	5.7	2.8
HCT116	6.4	2.6

## References

[1] Delmas D., Jannin B., Latruffe N. (2005). Resveratrol: Preventing properties against vascular alterations and ageing. Mol. Nutr. Food Res..

[2] Delmas D., Lancon A., Colin D., Jannin B., Latruffe N. (2006). Resveratrol as a chemopreventive agent: A promising molecule for fighting cancer. Curr. Drug Targets.

[3] Delmas D., Xiao J. (2012). Natural Polyphenols Properties: Chemopreventive and Chemosensitizing Activities. Anticancer Agents Med. Chem..

[4] Jang M., Cai L., Udeani G.O., Slowing K.V., Thomas C.F., Beecher C.W., Fong H.H., Farnsworth N.R., Kinghorn A.D., Mehta R.G. (1997). Cancer chemopreventive activity of resveratrol, a natural product derived from grapes. Science.

[5] Colin D., Gimazane A., Lizard G., Izard J.C., Solary E., Latruffe N., Delmas D. (2009). Effects of resveratrol analogs on cell cycle progression, cell cycle associated proteins and 5fluoro-uracil sensitivity in human derived colon cancer cells. Int. J. Cancer.

[6] Delmas D., Rebe C., Lacour S., Filomenko R., Athias A., Gambert P., Cherkaoui-Malki M., Jannin B., Dubrez-Daloz L., Latruffe N. (2003). Resveratrol-induced apoptosis is associated with Fas redistribution in the rafts and the formation of a death-inducing signaling complex in colon cancer cells. J. Biol. Chem..

[7] Sun Z.J., Pan C.E., Liu H.S., Wang G.J. (2002). Anti-hepatoma activity of resveratrol in vitro. World J. Gastroenterol..

[8] Fuggetta M.P., D’Atri S., Lanzilli G., Tricarico M., Cannavo E., Zambruno G., Falchetti R., Ravagnan G. (2004). In vitro antitumour activity of resveratrol in human melanoma cells sensitive or resistant to temozolomide. Melanoma Res..

[9] Delmas D., Rebe C., Micheau O., Athias A., Gambert P., Grazide S., Laurent G., Latruffe N., Solary E. (2004). Redistribution of CD95, DR4 and DR5 in rafts accounts for the synergistic toxicity of resveratrol and death receptor ligands in colon carcinoma cells. Oncogene.

[10] Colin D., Limagne E., Jeanningros S., Jacquel A., Lizard G., Athias A., Gambert P., Hichami A., Latruffe N., Solary E. (2011). Endocytosis of resveratrol via lipid rafts and activation of downstream signaling pathways in cancer cells. Cancer Prev. Res. (Phila).

[11] Breedveld P., Pluim D., Cipriani G., Dahlhaus F., van Eijndhoven M.A., de Wolf C.J., Kuil A., Beijnen J.H., Scheffer G.L., Jansen G. (2007). The effect of low pH on breast cancer resistance protein (ABCG2)-mediated transport of methotrexate, 7-hydroxymethotrexate, methotrexate diglutamate, folic acid, mitoxantrone, topotecan, and resveratrol in in vitro drug transport models. Mol. Pharmacol..

[12] Henry C., Vitrac X., Decendit A., Ennamany R., Krisa S., Merillon J.M. (2005). Cellular uptake and efflux of trans-piceid and its aglycone trans-resveratrol on the apical membrane of human intestinal Caco-2 cells. J. Agric. Food Chem..

[13] Cooray H.C., Janvilisri T., Van Veen H.W., Hladky S.B., Barrand M.A. (2004). Interaction of the breast cancer resistance protein with plant polyphenols. Biochem. Biophys. Res. Commun..

[14] Ramirez-Garza S.L., Laveriano-Santos E.P., Marhuenda-Munoz M., Storniolo C.E., Tresserra-Rimbau A., Vallverdu-Queralt A., Lamuela-Raventos R.M. (2018). Health Effects of Resveratrol: Results from Human Intervention Trials. Nutrients.

[15] Aires V., Limagne E., Cotte A.K., Latruffe N., Ghiringhelli F., Delmas D. (2013). Resveratrol metabolites inhibit human metastatic colon cancer cells progression and synergize with chemotherapeutic drugs to induce cell death. Mol. Nutr. Food Res..

[16] Delmas D., Passilly-Degrace P., Jannin B., Malki M.C., Latruffe N. (2002). Resveratrol, a chemopreventive agent, disrupts the cell cycle control of human SW480 colorectal tumor cells. Int. J. Mol. Med..

[17] Baarine M., Thandapilly S.J., Louis X.L., Mazue F., Yu L., Delmas D., Netticadan T., Lizard G., Latruffe N. (2011). Pro-apoptotic versus anti-apoptotic properties of dietary resveratrol on tumoral and normal cardiac cells. Genes Nutr..

[18] Aires V., Delmas D., Le Bachelier C., Latruffe N., Schlemmer D., Benoist J.F., Djouadi F., Bastin J. (2014). Stilbenes and resveratrol metabolites improve mitochondrial fatty acid oxidation defects in human fibroblasts. Orphanet J. Rare Dis..

[19] Mahyar-Roemer M., Katsen A., Mestres P., Roemer K. (2001). Resveratrol induces colon tumor cell apoptosis independently of p53 and precede by epithelial differentiation, mitochondrial proliferation and membrane potential collapse. Int. J. Cancer.

[20] Yuan L., Zhang Y., Xia J., Liu B., Zhang Q., Liu J., Luo L., Peng Z., Song Z., Zhu R. (2015). Resveratrol induces cell cycle arrest via a p53-independent pathway in A549 cells. Mol. Med. Rep..

[21] Chow S.E., Wang J.S., Chuang S.F., Chang Y.L., Chu W.K., Chen W.S., Chen Y.W. (2010). Resveratrol-induced p53-independent apoptosis of human nasopharyngeal carcinoma cells is correlated with the downregulation of DeltaNp63. Cancer Gene Ther..

[22] Sinicrope F.A., Hart J., Brasitus T.A., Michelassi F., Lee J.J., Safa A.R. (1994). Relationship of P-glycoprotein and carcinoembryonic antigen expression in human colon carcinoma to local invasion, DNA ploidy, and disease relapse. Cancer.

[23] Doyle L.A., Ross D.D. (2003). Multidrug resistance mediated by the breast cancer resistance protein BCRP (ABCG2). Oncogene.

[24] Wang L., Wang C., Jia Y., Liu Z., Shu X., Liu K. (2016). Resveratrol Increases Anti-Proliferative Activity of Bestatin Through Downregulating P-Glycoprotein Expression Via Inhibiting PI3K/Akt/mTOR Pathway in K562/ADR Cells. J. Cell. Biochem..

[25] Khan M., Maryam A., Mehmood T., Zhang Y., Ma T. (2015). Enhancing Activity of Anticancer Drugs in Multidrug Resistant Tumors by Modulating P-Glycoprotein through Dietary Nutraceuticals. Asian Pac. J. Cancer Prev..

[26] Khaleel S.A., Al-Abd A.M., Ali A.A., Abdel-Naim A.B. (2016). Didox and resveratrol sensitize colorectal cancer cells to doxorubicin via activating apoptosis and ameliorating P-glycoprotein activity. Sci. Rep..

[27] Wang Z., Zhang L., Ni Z., Sun J., Gao H., Cheng Z., Xu J., Yin P. (2015). Resveratrol induces AMPK-dependent MDR1 inhibition in colorectal cancer HCT116/L-OHP cells by preventing activation of NF-kappaB signaling and suppressing cAMP-responsive element transcriptional activity. Tumour Biol..

[28] Michalak K., Wesolowska O. (2012). Polyphenols counteract tumor cell chemoresistance conferred by multidrug resistance proteins. Anti-Cancer Agents Med. Chem..

[29] Farabegoli F., Papi A., Bartolini G., Ostan R., Orlandi M. (2010). (-)-Epigallocatechin-3-gallate downregulates Pg-P and BCRP in a tamoxifen resistant MCF-7 cell line. Phytomedicine.

[30] van Zanden J.J., Wortelboer H.M., Bijlsma S., Punt A., Usta M., Bladeren P.J., Rietjens I.M., Cnubben N.H. (2005). Quantitative structure activity relationship studies on the flavonoid mediated inhibition of multidrug resistance proteins 1 and 2. Biochem. Pharmacol..

[31] Delmas D., Aires V., Colin D.J., Limagne E., Scagliarini A., Cotte A.K., Ghiringhelli F. (2013). Importance of lipid microdomains, rafts, in absorption, delivery, and biological effects of resveratrol. Ann N. Y. Acad. Sci..

[32] Fang Y., Liang F., Liu K., Qaiser S., Pan S., Xu X. (2018). Structure characteristics for intestinal uptake of flavonoids in Caco-2 cells. Food Res. Int..

[33] Mieszala K., Rudewicz M., Gomulkiewicz A., Ratajczak-Wielgomas K., Grzegrzolka J., Dziegiel P., Borska S. (2018). Expression of genes and proteins of multidrug resistance in gastric cancer cells treated with resveratrol. Oncol. Lett..

